# The predictive power of ^18^F-FDG PET/CT two-lesions radiomics and conventional models in classical Hodgkin’s Lymphoma: a comparative retrospectively-validated study

**DOI:** 10.1007/s00277-025-06190-8

**Published:** 2025-01-14

**Authors:** Elizabeth Katherine Anna Triumbari, David Morland, Roberto Gatta, Luca Boldrini, Marco De Summa, Silvia Chiesa, Annarosa Cuccaro, Elena Maiolo, Stefan Hohaus, Salvatore Annunziata

**Affiliations:** 1 Department of Radiology, Radiotherapy and Hematology, Fondazione Policlinico Universitario A. Gemelli IRCCS, Rome, Italy; 2 Department of Radiological Sciences and Hematology, Università Cattolica del Sacro Cuore, Rome, Italy; 3 Institut Godinot and CReSTIC EA 3804, Université de Reims Champagne-Ardenne, Reims, France; 4 Department of Clinical and Experimental Sciences, University of Brescia, Brescia, Italy; 5 Medipass S.p.a. Integrative Service, Rome, Italy; 6 Department of Radiology, Radiotherapy and Hematology, Unità di Medicina Nucleare, GSTeP Radiopharmacy, Fondazione Policlinico Universitario A.Gemelli IRCCS, Rome, Italy

**Keywords:** Hodgkin, ^18^F-FDG, PET/CT, Radiomics, Prediction

## Abstract

In a previous preliminary study, radiomic features from the largest and the hottest lesion in baseline ^18^F-FDG PET/CT (bPET/CT) of classical Hodgkin’s Lymphoma (cHL) predicted early response-to-treatment and prognosis. Aim of this large retrospectively-validated study is to evaluate the predictive role of two-lesions radiomics in comparison with other clinical and conventional PET/CT models. cHL patients with bPET/CT between 2010 and 2020 were retrospectively included and randomized into training-validation sets. Target lesions were: Lesion_A, with largest axial diameter (D_max_); Lesion_B, with highest SUV_max_. Total-metabolic-tumor-volume (TMTV) was calculated and 212 radiomic features were extracted. PET/CT features were harmonized using ComBat across two scanners. Outcomes were progression-free-survival (PFS) and Deauville Score at interim PET/CT (DS). For each outcome, three predictive models and their combinations were trained and validated: - radiomic model “R”; - conventional PET/CT model “P”; - clinical model “C”. 197 patients were included (training = 118; validation = 79): 38/197 (19%) patients had adverse events and 42/193 (22%) had DS ≥ 4. In the training phase, only one radiomic feature was selected for PFS prediction in model “R” (Lesion_B *F_cm.corr*, C-index 66.9%). Best “C” model combined stage and IPS (C-index 74.8%), while optimal “P” model combined TMTV and D_max_ (C-index 63.3%). After internal validation, “C”, “C + R”, “R + P” and “C + R + P” significantly predicted PFS. The best validated model was “C + R” (C-index 66.3%). No model was validated for DS prediction. In this large retrospectively-validated study, a combination of baseline ^18^F-FDG PET/CT two-lesions radiomics and other conventional models showed an added prognostic power in patients with cHL. As single models, conventional clinical parameters maintain their prognostic power, while radiomics or conventional PET/CT alone seem to be sub-optimal to predict survival.

**Supplementary Information:**

The online version contains supplementary material available at 10.1007/s00277-025-06190-8.

## Introduction

Hodgkin’s Lymphoma (HL) is a B-cell lymphoid malignancy with an incidence of 2.3–2.9/100.000 people/year [1], and two main histological types: classical HL (cHL, 95% of HL cases) and nodular lymphocyte-predominant (5%) [1, 2]. The five-year survival rate varies considerably, mostly depending on disease stage at diagnosis [3] assessed by baseline Fluorine-18-Fluorodeoxiglucose (^18^F-FDG) Positron Emission Tomography/Computed Tomography (bPET/CT) [1, 4–6]. bPET/CT enables patients’ risk stratification, therapeutic strategy planning [2, 5] and comparison for response-to-treatment assessment [5]. Other parameters used for risk stratification at baseline are disease stage, presence of bulky disease and the International Prognostic Score (IPS) [1, 7]. At interim PET/CT (iPET/CT), performed after 2–4 courses of primary chemotherapy (PCT), the 5-point Deauville Score (DS) proved high prognostic efficacy [8, 9], guiding physicians to decide whether to complete chosen therapies, add or omit treatment components [2].

Despite strategic use of DS and advances in treatment, about 20–30% of HL patients still relapse or die because of disease progression [1, 10]. Thus, clinical risk-stratification parameters need to be implemented, and earlier prognostic predictors need to be identified at baseline. Increasing interest has grown towards semiquantitative PET parameters as SUVmax, Total Metabolic Tumor Volume (TMTV) [11], and more recently higher-order radiomic features and topographic dissemination features [12, 13].

Differently from other lymphoma sub-types [12], few studies have investigated higher-order target lesion radiomics in HL, attempting histological classification [14] and prediction of staging [15, 16], early metabolic response [17, 18] or refractory disease [19, 20]. Innovative methodological frameworks have also been proposed [16, 21]. In a recent preliminary study, we found that cHL PET/CT radiomic features from two easily recognizable target lesions (the largest and the hottest) may provide relevant information in terms of early response-to-treatment and prognosis [16]. However, evidence on cHL PET/CT radiomics is still sub-optimal because of the need of methodological harmonization, in terms of selection of target volumes, numerosity of study cohorts, internal and external validation, and comparison with conventional clinical and PET/CT predictive models [22, 23].

Therefore, our study aimed at investigating the predictive role of baseline ^18^F-FDG-PET/CT (bPET/CT) two-lesions radiomics in comparison with other clinical and conventional PET/CT prognostic models, in a large retrospectively validated study in classical Hodgkin’s Lymphoma (cHL).

## Methods

### Study design and data collection

This retrospective monocentric study has been carried out in accordance with The Code of Ethics of the World Medical Association (Declaration of Helsinki) and was approved by the institutional Ethics Committee (ID 3834). Included subjects signed an informed consent form.

Medical records of patients consecutively diagnosed with HL and referred to the Hematology Unit between September 2010 and January 2020 were reviewed. Patients were included if they had an on-site bPET/CT and were ≥ 18 years old. Exclusion criteria were: no information available after diagnosis, nodular lymphocyte-predominant HL histology [2], other synchronous/metachronous tumors, extensive surgical resection of HL disease for diagnostic purposes before bPET/CT, first evaluation at disease relapse.

Age at diagnosis, sex, histology subtype, Ann Arbor Stage, presence of bulky disease, International Prognostic Score for HL (IPS), PET/CT scanner on which the bPET/CT was performed, DS at iPET after two cycles of PCT and the latest date of follow-up clinical observation were recorded. Ann Arbor stage and bulk status were used to classify disease stage as early (Ann Arbor stages IA to IIB without bulk) or advanced (Ann Arbor stages IIB with bulk to IV) [1].

### PET/CT acquisition protocol

PET/CT studies were acquired according to the EANM/EARL accreditation guidelines [24]. Patients fasted for ≥ 6 h and their blood glucose levels were < 200 mg/dl before administration of 236 ± 50 MBq (6.37 ± 1.35 mCi) of ^18^F-FDG. Images were acquired after 60 ± 10 min of uptake time using a Gemini GXL (Philips Healthcare) or a Biograph mCT (Siemens Healthineers) PET/CT scanner, applying their standard reconstruction protocols (Supplemental Table 1).

### PET/CT and radiomic features

PET/CT images were reviewed by two experienced nuclear medicine physicians, blinded to patients’ clinical and follow-up data.

**Fig. 1:**
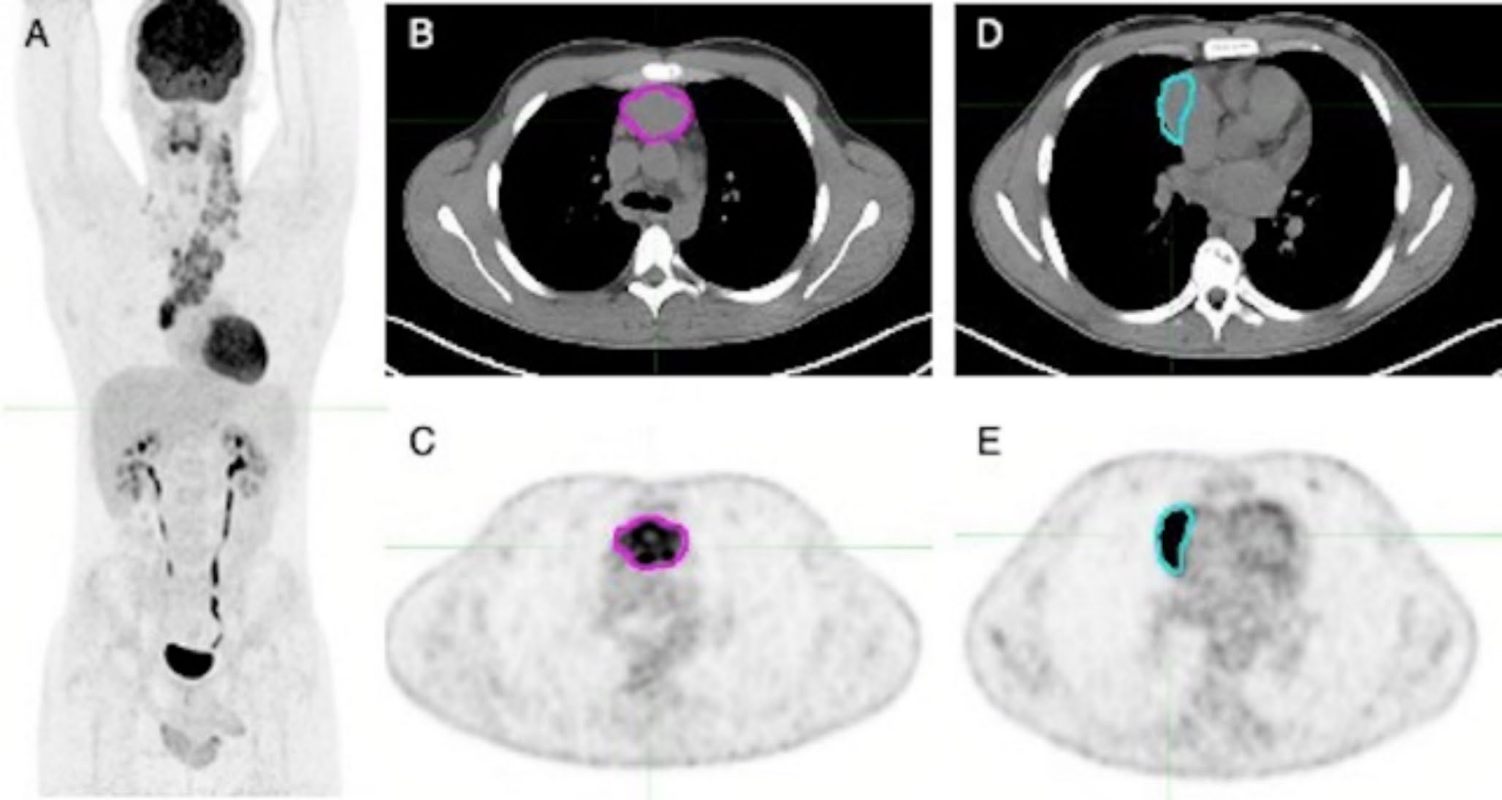
Target lesion contouring with PET_Edge_ tool from MIM Encore Software (version 7.0.5 MIM Encore Software Inc., Cleveland, OH). (**A**) Maximum-intensity projection of an 18-year-old male patient with Stage IIA nodular sclerosing Hodgkin’s Lymphoma. Axial CT (**B**) and PET (**C**) views of Lesion_A (pink contoured VOI, D_max_ = 4.33 cm). Axial CT (**D**) and PET (**E**) views of Lesion_B (cyan contoured VOI, SUV_max_ = 11.81)

For target lesion contouring, a semiautomatic gradient-based segmentation tool (PET_Edge_, version 7.0.5 MIM Encore Software Inc., Cleveland, OH) with no manual adjustments was used to delineate volumes of interest (VOIs) around two nodal lesions for each bPET/CT scan [25, 26]. As previously described [16], Target Lesion_A was the lymph node lesion with the largest axial diameter (D_max_) identified on CT images. When a bulky tumor was present, Lesion_A was identified as the single distinguishable lesion with D_max_ among contiguous lesions in the bulk and its D_max_ was measured (Fig. 1). Target Lesion_B was the lymph node lesion with the highest maximum Standardized Uptake Value (SUV_max_). When more than one lesion visually showed similar ^18^F-FDG uptake, a VOI was drawn around each one to choose the hottest and its SUV_max_ was recorded. According to these definitions, Lesion_A and Lesion_B could correspond to the same lesion.

**Fig. 2:**
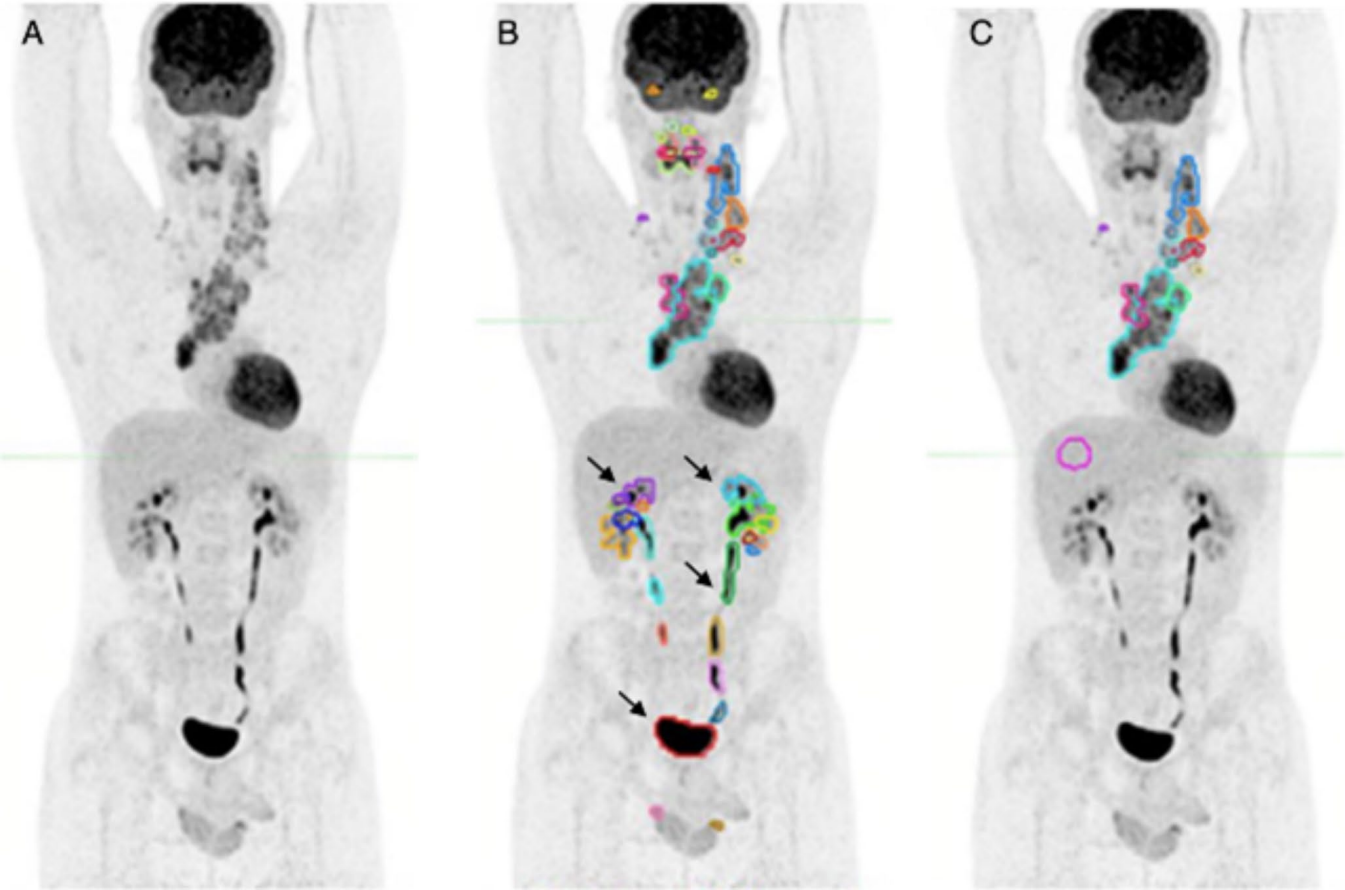
Total Metabolic Tumor Volume (TMTV) contouring with LesionID tool from MIM Encore Software (version 7.0.5 MIM Encore Software Inc., Cleveland, OH). (**A**) Maximum-intensity projection of an 18-year-old male patient with Stage IIA nodular sclerosing Hodgkin’s Lymphoma. (**B**) Semiautomatic threshold-based segmentation of the whole body, comprising some physiological sites of uptake (e.g., arrows), later excluded from the computation. (**C**) Revised lesion contouring, then processed by the software to obtain TMTV volumetric parameter (149,08 ml); pink circle: region of interest on the right liver lobe for PERCIST-based cut-off criterion for volumes of interest determination

For total metabolic tumor volume (TMTV), a whole-body PET segmentation tool (LesionID, version 7.0.5 MIM Encore Software Inc., Cleveland, OH) was applied to each bPET/CT scan. The program workflow firstly used a PET Response Criteria in Solid Tumors (PERCIST)-based background threshold (liver) to identify all lesions with higher uptake, then applied a fixed relative threshold of ≥ 41% of the SUV_max_ of each VOI to create boundaries of the metabolically active region within each lesion [27]. Physicians were required to reject false-positive lesions (physiological uptake, external contamination, lymphoma-unrelated) (Fig. 2).

DICOM and corresponding RT-struct files of the contoured target lesions were then subjected to the extraction of radiomic features using Moddicom [28, 29], an open-source software library in R [30], Image Biomarker Standardization Initiative (IBSI)-compliant [31]. Features belonged to the following IBSI classes: Morphological, Intensity-based statistical, Intensity-histogram, Grey-level co-occurrence matrix (GLCM), Grey-level run-length matrix (GLRLM), Grey-level size-zone matrix (GLSZM) [31] (Supplemental Table 2). No spatial interpolation or kernel-based filter was needed due to the homogeneous geometry in the DICOM series [31].

### Statistical analysis and predictive models

Statistical analyses were conducted using R software [30] and XLSTAT 2022.1.2 (Addinsoft). For descriptive statistics, mean and standard deviation were used for quantitative variables, while absolute number and percentage were adopted for binary variables. Fisher’s exact test or t-test were used to compare the groups when appropriate.

#### Randomization and imputation

**Fig. 3:**
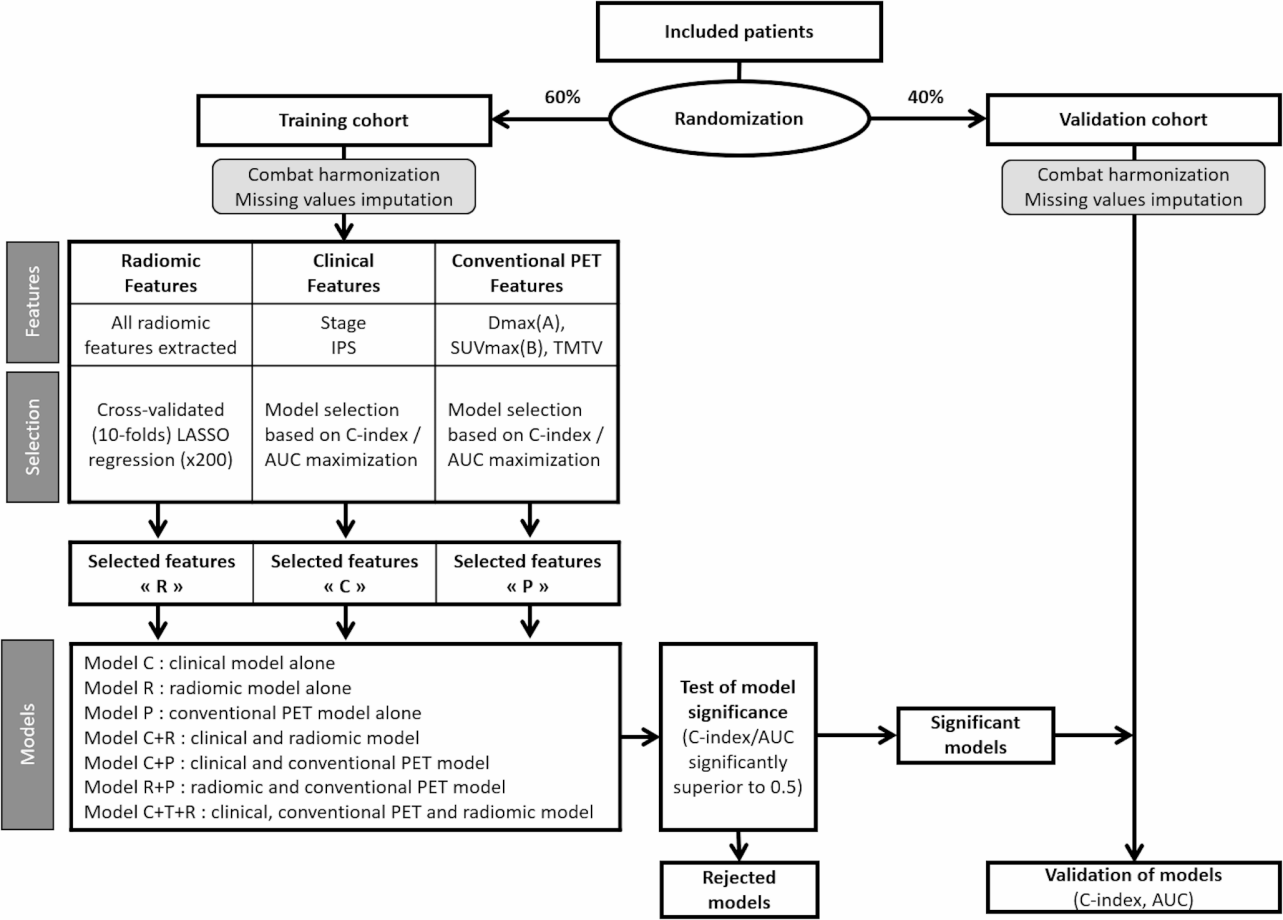
Statistical workflow

Included patients were randomized into two groups (60% training, 40% validation). As previously described [32], ComBat harmonization was performed for all bPET/CT features, using the scanner with the latest technology as reference. Missing features were then imputed using mean values (Fig. 3).

#### PFS prediction

PFS was defined as the interval between cHL histological diagnosis and the first clinical detection of adverse events, defined as progression during treatment, lack of complete remission after PCT or disease relapse. Radiomic features for PFS prediction were selected using Cox models and LASSO regression on the training cohort. The optimal penalty coefficient λ was determined using 200 repetitions of a 10-fold cross-validated LASSO regression. Using this λ, relevant radiomic features were identified and combined in a Cox radiomic model “R”. Two features were considered among clinical parameters (IPS ≥ 3 and advanced stage). The optimal Cox clinical model “C” (IPS alone, stage alone, both) was identified based on C-index maximization. D_max_ from Lesion_A, SUV_max_ from Lesion_B, and TMTV were considered as conventional bPET/CT parameters. The optimal Cox conventional PET/CT model “P” was identified based on C-index maximization. Composite models were then created using all combinations of “R”, “C” and “P” (Fig. 3). C-index (value and 95% confidence intervals, 95%CI) was calculated for each model. Models were considered significant if C-index was significantly different from 50% (CI not including 50%, random chance). Finally, significant models were applied to the validation cohort (Fig. 3).

#### DS prediction

DS at iPET/CT was considered as a binary outcome: good response with DS 1 to 3 vs. insufficient response with DS 4 and 5 [5]. To predict DS, a new feature identification and model selection was performed on the training cohort, using the same randomization. Patients with a missing DS were excluded. A similar workflow to that used for the PFS outcome was applied, Area Under the Curve (AUC) and binomial parametrization being used instead of C-index metrics and Cox models (Fig. 3). Models were considered significant if AUC was significantly different from 50% (CI not including 50%, low accuracy). Finally, significant models were applied to the validation cohort (Fig. 3).

## Results

### Study cohort, PET/CT and radiomic features

**Fig. 4:**
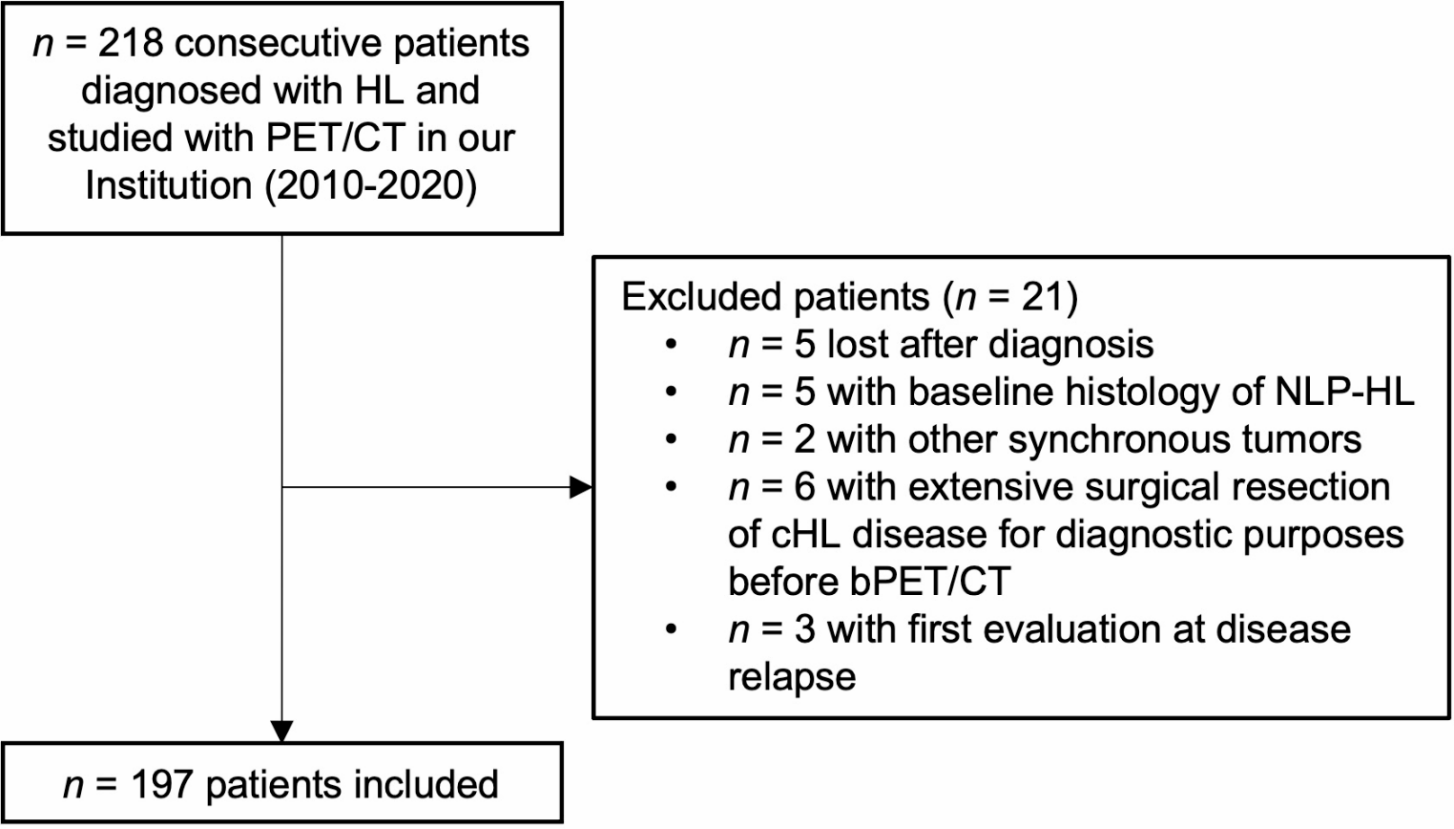
Flowchart of patients’ selection. NLP-HL = Nodular Lymphocyte Predominant Hodgkin’s Lymphoma

Among the 218 HL patients referred to the Hematology Unit, 197 fulfilled the inclusion criteria and were included (Fig. 4). The first two cycles of PCT consisted of Adriamycin, Bleomycin, Vinblastine and Dacarbazine (ABVD) for all patients. DS-guided therapy escalation was performed in 19/197 (9.6%) patients and 161/197 (81.7%) underwent involved-field radiotherapy after PCT. The mean follow-up time was 53.8 ± 29.8 months. Overall, 42/193 (21.7%) patients had iPET/CT DS ≥ 4 and 38/197 (19.3%) patients had adverse events at follow-up.

Table 1 shows training (*n =* 118) and validation (*n =* 79) cohorts’ characteristics. Lesion_A and Lesion_B corresponded in 70/118 patients (59%) in the training cohort and in 41/79 patients (52%) in the validation cohort (*p =* 0.31). D_max_, SUV_max_ and TMTV values after ComBat harmonization are available in Table 1, as well as PFS and DS data. For each target lesion, Moddicom extracted 212 radiomic features. Imputation was needed for 138 features for the training cohort [138/(212 features×2 lesions×118 patients) = 0.27%] and for 155 features in the validation cohort [155/(212 × 2 × 79) = 0.46%].

**Table 1:** Patients’ characteristics (*n* = 197)

Characteristics	Training dataset *n =* 118	Validation dataset *n =* 79	Comparison *p*-value
Age at diagnosis (years)			
Mean ± SD	38.9 ± 17.1	41.7 ± 18.0	0.28
Sex (*n*)			
Male	50 (42.4%)	44 (55.7%)	0.08
Female	68 (57.6%)	35 (44.3%)	
Histology subtype (*n*)			
Nodular sclerosing	92 (78.0%)	66 (83.5%)	
Mixed cellularity	6 (5.1%)	4 (5.1%)	
Lymphocyte-rich	3 (2.5%)	2 (2.5%)	0.85
Lymphocyte-depleted	2 (1.7%)	1 (1.3%)	
Classical, not specified	15 (12.7%)	6 (7.6%)	
Ann Arbor Stage (*n*)			
I	2 (1.7%)	1 (1.3%)	
II	60 (50.8%)	38 (48.1%)	0.89
III	22 (18.6%)	13 (16.5%)	
IV	34 (28.8%)	27 (34.2%)	
Bulk (*n*)			
Yes	27 (22.9%)	16 (20.3%)	0.73
No	91 (77.1%)	63 (79.7%)	
Stage* (*n*)			
Early	58 (49.2%)	33 (41.8%)	0.38
Advanced	60 (50.8%)	46 (58.2%)	
IPS (*n*)			
<3	79 (66.9%)	51 (64.6%)	0.76
≥3	39 (33.1%)	28 (35.4%)	
PET/CT system (*n*)			
Gemini GXL	59 (50.0%)	38 (48.1%)	0.89
Biograph mCT	59 (50.0%)	41 (51.9%)	
D_max_ (Lesion_A) (cm)			
Mean ± SD [Range]	4.4 ± 1.9 [0.8–10.9]	4.7 ± 2.2 [1.4–11.1]	0.12
After ComBat	4.4 ± 1.9 [1.0-10.9]	4.5 ± 2.0 [1.4–10.0]	0.56
SUV_max_ (Lesion_B)			
Mean ± SD [Range]	15.3 ± 7.2 [2.6–47.2]	17.5 ± 9.2 [5.9–66.0]	0.06
After ComBat	17.6 ± 6.0 [4.9–44.2]	19.1 ± 7.7 [7.3–55.9]	0.23
TMTV (ml)			
Mean ± SD [Range]	233.1 ± 231.7 [1.4-1075.3]	228.7 ± 254.4 [0.7-1119.8]	0.89
After ComBat	238.9 ± 222.8 [1.4-1018.9]	244.7 ± 255.7 [9.3-1119.8]	0.86
Interim Deauville Score (*n*)			
1–3	86 (72.9%)	65 (82.3%)	0.19
4–5	30 (25.4%)	12 (15.2%)	
Missing	2 (1.7%)	2 (2.5%)	
Progression Free Survival (*n*)			
Adverse events	25 (21.2%)	13 (16.5%)	0.36
Censored	93 (78.8%)	66 (83.5%)	

D_max_: largest maximum axial diameter of the largest lesion (Lesion_A); IPS: International Prognostic Score for Hodgkin’s Lymphoma; SD: standard deviation; Stage*: early if Ann Arbor stages I to IIA with no bulk, advanced if Ann Arbor stages IIB with bulk to IV; SUV_max_: maximal Standard Uptake Value of the hottest lesion (Lesion_B); TMTV: Total Metabolic Tumor Volume

### Predictive models

#### PFS prediction

In the training cohort, only one radiomic feature was relevant for PFS prediction (*F_cm.corr*. from Lesion_B, C-index 66.9% [56.3–77.5], model “R”). Optimal clinical model “C” combined stage and IPS (C-index 74.8% [66.0-83.6]). Optimal conventional PET/CT model “P” combined TMTV and D_max_ (C-index 63.3% [51.5–75.1]) (Table 2). Models “C”, “C + R”, “R + P” and “C + R + P” remained significant at the validation phase. The best model to predict PFS was “C + R”, with C-index 66.3% [55.9–76.7] (Table 2). Similar results were obtained in a sub-group analysis, performed for completeness of information, only on patients with overlapping Lesion_A and Lesion_B in the validation cohort (Supplemental Table 3).

**Table 2:** Clinical, radiomic, conventional PET/CT and combined models’ significance for PFS prediction

Models	C-index [95% CI]	
	**Training (*n =*** **118)**	**Validation (*n =*** **79)**
C	74.8% [66.0;83.6]*	64.7% [54,1;75.3]*
R	66.9% [56.3;77.5]*	55.9% [44.9;66.9]
P	63.3% [51.5;75.1]*	59.9% [49.1;70.7]
C + R	78.8% [70.0;87.6]*	66.3% [55.9;76.7]*
C + P	77.1% [67.1;87.1]*	57.8% [46.8;68.8]
R + P	70.1% [60.3;79.9]*	61.6% [50.8;72.4]*
C + R + P	79.6% [70.6;88.6]*	60.9% [50.1;71.7]*

C: clinical model (stage and IPS); P: conventional PET/CT model (D_max_ of Lesion_A and TMTV); R: radiomic model (*F_cm.corr* from Lesion_B); *: significant models

#### DS prediction

In the training cohort, three relevant radiomic features were extracted for model “R”, with an AUC of 75.3% [64.4–86.3]: *F_szm.glnu* and *F_rlm.2.5D.rlnu* from Lesion_B, *F_szm.glnu* from Lesion_A, Optimal model “C” combined stage and IPS (AUC 66.3% [55.6–77.1]). Model “P” combined TMTV, D_max_ and SUV_max_ (AUC 71.0% [59.3–82.6]). In the validation set, no model remained significant (Table 3).

**Table 3:** Clinical, radiomic, conventional PET/CT and combined models’ significance for DS prediction

Models	AUC [95% CI]	
	**Training (*n =*** **116)**	**Validation (*n =*** **77)**
C	66.3% [55.6;77.1]*	60.9% [45.8;76.0]
R	75.3% [64.4;86.3]*	55.3% [40.1;70.4]
P	71.0% [59.3;82.6]*	63.6% [48.2;79.0]
C + R	77.2% [66.4;88.0]*	55.9% [39.7;72.1]
C + P	72.9% [61.0;84.8]*	62.9% [46.6;79.3]
R + P	82.2% [74.2;90.3]*	53.2% [33.6;72.8]
C + R + P	82.9% [74.4;91.4]*	55.0% [36.0;74.0]

C: clinical model (stage and IPS); P: conventional PET/CT model (D_max_ of Lesion_A, SUV_max_ of Lesion_B and TMTV); R: radiomic model (*F_szm.glnu* and *F_rlm.2.5D.rlnu* from Lesion_B and *F_szm.glnu* from Lesion_A); *: significant models

Different results were found in the sub-group analysis, performed for completeness of information, on patients with overlapping Lesion_A and Lesion_B in the validation cohort (Supplemental Table 4), where model “C” and all combination models resulted significant. Since this is was sub-analysis, carried out on a very small number of patients (only 41), and outside the initial scope of the study, these results represent a starting point for future scientific speculations.

## Discussion

In this comparative retrospectively validated study, a combination of baseline ^18^F-FDG PET/CT two-lesions radiomics and other conventional prognostic models showed a predictive power in patients with cHL. As single models, radiomics or conventional PET/CT alone seem to be sub-optimal to predict survival, while conventional clinical parameters maintain their prognostic power.

### Radiomics targets

Lymphoma often lacks a “primary lesion”, presenting as a systemic malignancy with high heterogeneity. According to our previous preliminary study [16], lesions with the largest diameter (D_max_) and highest SUV_max_ can translate the growth-capability (size) and the biological aggressiveness (metabolic activity) in cHL, respectively. Moreover, they are easily and reproducibly identifiable in PET/CT images. Indeed, though there is a common belief that a 3D measurement of tumor lesions should be pursued, no study has shown superlative results, and D_max_ and SUV_max_ are still recognized as the standard of practice [33, 34]. These two parameters were suggested as a possible compromise in another HL study [21] and were recently adopted for bPET/CT radiomics in Diffuse Large B Cell Lymphoma [35]. Other HL studies suggested the use of radiomics from bulky lesion [17] or from all lesions [19, 21]. The use of the bulky lesion would have been comparable to the use of the single lesion with largest D_max_ in our work, but with the disadvantage of segmenting a large heterogeneous tissue that might have underestimated the volume and influence texture measurements [21, 36, 37]. Radiomics of all lymphoma lesions has been experimented with interesting results [19, 21], but it would imply a hardly implementable and interpretable procedure in the clinical practice, even though the use of whole-body semi-automatic segmentation methods for TMTV calculation have been already proposed [11]. Therefore, a suitable trade-off between one-lesion radiomics and all-lesions radiomics could be useful to improve the use of in cHL clinical practice [16, 21].

### PFS prediction

**Fig. 5:**
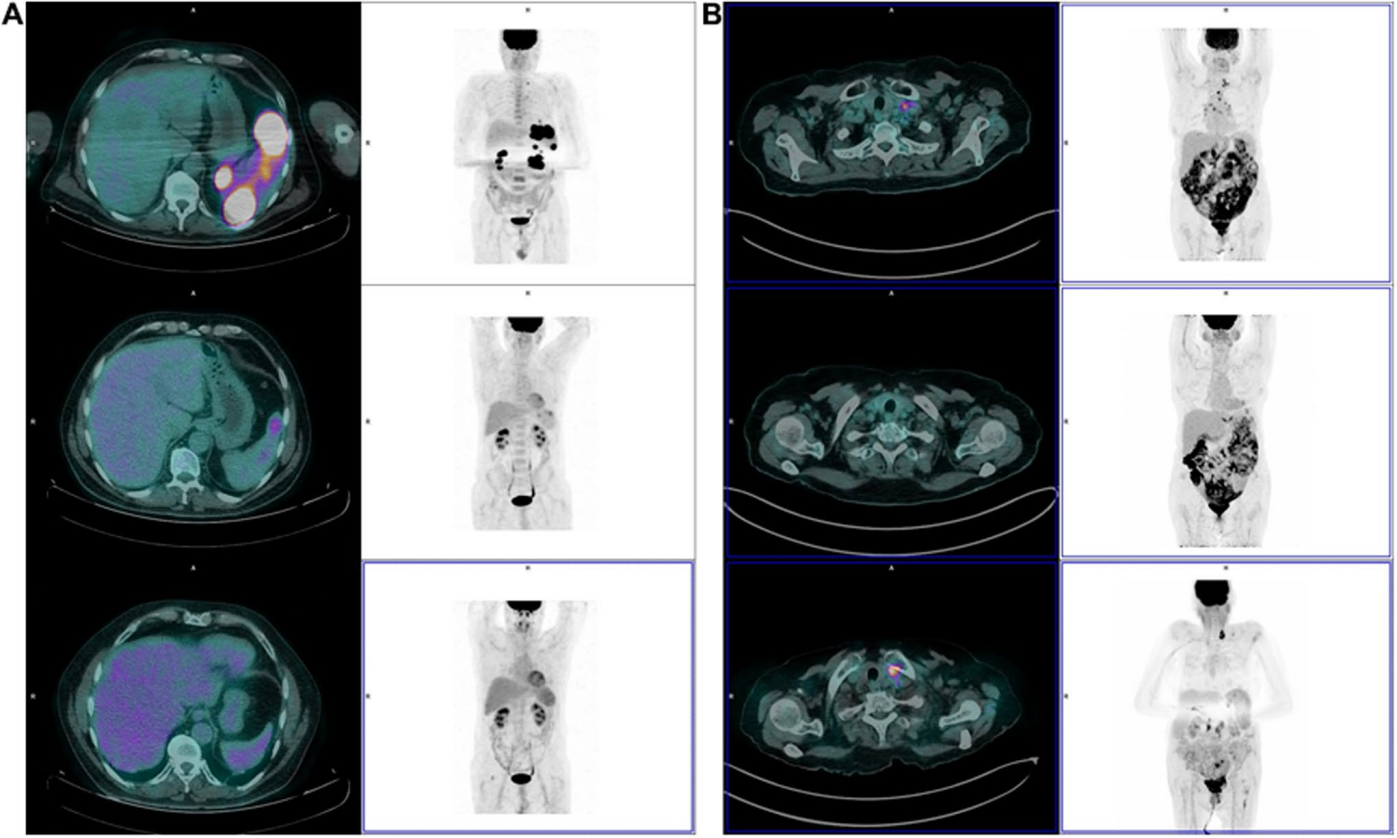
Baseline and further PET exams of (**A**) Patient 1 (Stage 4, IPS 3, bPET in **A**), who had a low F_cm.corr and still experienced no event at 79.8 months of follow-up; (**B**) Patient 2 (Stage 3, IPS 4, bPET in **B**) had a high value of F_cm.corr and progressed after 5.1 months of follow-up

According to our results, the radiomic model “R” predicted PFS in the training cohort but did not maintain its prognostic value in the validation set (Table 2). Only F_cm.corr feature derived from the hottest lesion (Lesion_B) was selected, measuring the linear dependency of grey levels of neighboring pixels [38] (Supplemental Table 2). Even if it did not maintain its significancy in the validation cohort, some attention was dedicated to this result, with interesting observations that could inspire future work: two patients with advanced stage disease cHL presented with an opposite magnitude of F_cm.corr feature at bPET, and two very different PFS values at follow-up. Patient 1 (Stage 4, IPS 3, bPET in Fig. 5A) had a low F_cm.corr and still experienced no event at 79.8 months of follow-up. Patient 2 (Stage 3, IPS 4, bPET in Fig. 5B) had a high value of F_cm.corr and progressed after 5.1 months of follow-up. Future studies on wider cohorts might find better predictive radiomic features for PFS, allowing anticipation of the decision-making regarding escalation or de-escalation of therapies.

D_max_ and TMTV (model “P”) were prognostic parameters in the training cohort but were not validated. Indeed, PET/CT volumetric parameters such as bulky disease and TMTV previously demonstrated their prognostic significance in cHL [1, 33, 39]. The clinical model was identified in the combination of stage and IPS, two parameters that are constantly present in the available panel of risk assessment [1, 6].

Combined models showed an added prognostic power at the validation stage. The best validated model was “C + R”, showing a C-index of 66.3%, comparable to the C-index of 64.7% of the “C” model alone. Thus, our combined clinical-radiomic model could consolidate the prognostic power of clinical or radiomic parameters alone. Given the routinary use of bPET/CT for cHL staging, the implementation of a radiomic model derived by bPET/CT could support and increase the predictive power already available with conventional clinical models [2, 12], as shown in other lymphoma sub-types [40].

Interestingly, the combined model “R + P” had a prognostic significance at validation, where single models alone failed. Moreover, SUV_max_ was not significant and so excluded for model “P” (Dmax + TMTV), demonstrating that hidden biological information could be provided by whole-lesion parameters, such as diameters, metabolic tumor burden and radiomics, over conventional single-voxel parameters (e.g. SUVmax). Moreover, whole-lesion features may be provided also by other biological parameters, such as CD68 and PDL1 expression, as expression of prevalent non-tumoral microenvironment in cHL lesions [2].

### DS prediction

At validation, no model was significant to predict DS (Table 3). The relevant radiomic features were *F_rlm.2.5.rlnu* from Lesion_A and *F_szm.glnu* from both Lesion_A and Lesion_B [38]. *F_rlm.2.5.rlnu* measures similarity of run lengths throughout the image [38]. *F_szm.glnu* measures similarity of gray-level intensity values in the image [38] (Supplemental Table 2). Interestingly, all models were more significant than the “C” model alone in the training set, but none of them was validated (Table 3). Thus, radiomic features from the largest Lesion_A seem to be more DS predictive than the hottest lesion, but further studies are needed to validate these findings. Possibly, DS prediction could be improved by evaluating other baseline parameters, such as non-tumoral microenvironment and genomics [2].

### Limitations and perspectives

As for input parameters, aim of this study was to evaluate the heterogeneity of ^18^F-FDG uptake in pathological target lesions, in comparison with other conventional predictive model. Evaluation of topographic dissemination was not in the aim of this study, even though they showed significant predictive power in literature [13]. Similarly, co-registered CT-based radiomics was not considered, except to extent with diameters. The downside of the use of two scanners was overcome by ComBat harmonization [32]. Data imputation was performed to preserve all cases, for a very low percentage of radiomic features (< 0.5%). Notably, the four features extracted through LASSO regression for PFS and DS prediction were never imputed.

As for output data, the division in training and validation sets resulted in smaller sample sizes and sub-optimal predictive power in the validation stage, even if our cohort is one of the largest monocentric for cHL with a reproducible internal validation [12, 23, 41, 42]. Our workflow could have been replaced by other validation methods (e.g., k-fold or cross-fold methods), but we optioned for independency of data and avoidance of data-leakage [22]. Moreover, considering the long follow-up time of our patients, the number of events recorded was rather low, yet expected [1, 2].

External validation studies will be needed to confirm our internally-validated results and spread the use of these models in clinical practice, as already proposed in a recent multicentric study [43].

## Conclusions

In this comparative retrospectively-validated study, a combination of baseline ^18^F-FDG PET/CT two-lesions radiomics and other conventional models showed an added prognostic power in patients with cHL. In a validation stage, conventional clinical parameters maintain their prognostic power, while radiomics or conventional PET/CT alone seem to be sub-optimal to predict survival.

Larger external validation studies are needed to confirm these results.

## Electronic supplementary material

Below is the link to the electronic supplementary material.

Supplementary Material 1

## Data Availability

Data is provided within the manuscript or supplementary information files.

## References

[1] Eichenauer DA, Aleman BMP, André M et al (2018) Hodgkin lymphoma: ESMO Clinical Practice guidelines for diagnosis, treatment and follow-up. Ann Oncol 29:iv19–iv29. 10.1093/annonc/mdy080 29796651 10.1093/annonc/mdy080

[2] Ansell SM, Hodgkin lymphoma (2020) A 2020 update on diagnosis, risk-stratification, and management. Am J Hematol 95:978–989. 10.1002/ajh.25856 10.1002/ajh.25856 32384177

[3] SEER*Explorer (2022) An interactive website for SEER cancer statistics [Internet]. Surveillance Research Program, National Cancer Institute. Accessed October 24. https://seer.cancer.gov/explorer/

[4] Barrington SF, Mikhaeel NG, Kostakoglu L et al (2014) Role of Imaging in the Staging and Response Assessment of Lymphoma: Consensus of the International Conference on Malignant Lymphomas Imaging Working Group. J Clin Oncol. 32:3048–3058. 10.1200/JCO.2013.53.5229 10.1200/JCO.2013.53.5229 PMC5015423 25113771

[5] Cheson BD, Fisher RI, Barrington SF et al (2014) Recommendations for initial evaluation, staging, and Response Assessment of Hodgkin and Non-hodgkin Lymphoma: the Lugano classification. J Clin Oncol 32:3059–3067. 10.1200/JCO.2013.54.8800 25113753 10.1200/JCO.2013.54.8800 PMC4979083

[6] Hoppe RT, Advani RH, Ai WZ et al (2022) January,. Hodgkin Lymphoma, Version 2.2022, NCCN Clinical Practice Guidelines in Oncology. J Natl Compr Canc Netw. Accessed https://www.nccn.org/professionals/physician_gls/pdf/hodgkins.pdf 10.6004/jnccn.2020.0026 32502987

[7] Hasenclever D, Diehl V, Armitage JO et al (1998) A prognostic score for Advanced Hodgkin’s Disease. N Engl J Med 339:1506–1514. 10.1056/NEJM199811193392104 9819449 10.1056/NEJM199811193392104

[8] Meignan M, Gallamini A, Meignan M et al (2009) Report on the First International Workshop on interim-PET scan in lymphoma. Leuk Lymphoma. 50:1257–1260. 10.1080/10428190903040048 10.1080/10428190903040048 19544140

[9] Barrington SF, Qian W, Somer EJ et al (2010) Concordance between four European centres of PET reporting criteria designed for use in multicentre trials in Hodgkin lymphoma. Eur J Nucl Med Mol Imaging 37:1824–1833. 10.1007/s00259-010-1490-5 20505930 10.1007/s00259-010-1490-5

[10] Engert A, Diehl V, Franklin J et al (2009) Escalated-dose BEACOPP in the treatment of patients with Advanced-Stage Hodgkin’s lymphoma: 10 years of Follow-Up of the GHSG HD9 study. J Clin Oncol 27:4548–4554. 10.1200/JCO.2008.19.8820 19704068 10.1200/JCO.2008.19.8820

[11] Driessen J, Zwezerijnen GJC, Schöder H et al (2022) The impact of semiautomatic segmentation methods on metabolic tumor volume, intensity, and dissemination Radiomics in 18F-FDG PET scans of patients with classical Hodgkin Lymphoma. J Nucl Med 63(9):1424–1430. 10.2967/jnumed.121.263067 34992152 10.2967/jnumed.121.263067 PMC9454468

[12] Rizzo A, Triumbari EKA, Gatta R et al (2021) The role of 18F-FDG PET/CT radiomics in lymphoma. Clin Transl Imaging 9:589–598. 10.1007/s40336-021-00451-y

[13] Albano D, Treglia G, Dondi F et al (2023) 18F-FDG PET/CT Maximum Tumor Dissemination (Dmax) in Lymphoma: A New Prognostic Factor? Cancers (Basel).; 26;15(9):2494. 10.3390/cancers15092494 10.3390/cancers15092494 PMC10177347 37173962

[14] Lippi M, Gianotti S, Fama A et al (2020) Texture analysis and multiple-instance learning for the classification of malignant lymphomas. Comput Methods Programs Biomed 185:105153. 10.1016/j.cmpb.2019.105153 31678792 10.1016/j.cmpb.2019.105153

[15] Lartizien C, Rogez M, Niaf E, Ricard F, Computer-Aided (2014) Staging of Lymphoma patients with FDG PET/CT imaging based on Textural Information. IEEE J Biomed Health Inf 18:946–955. 10.1109/JBHI.2013.2283658 10.1109/JBHI.2013.2283658 24081876

[16] Triumbari EKA, Gatta R, Maiolo E et al (2023) Baseline 18F-FDG PET/CT Radiomics in Classical Hodgkin’s lymphoma: the predictive role of the Largest and the hottest lesions. Diagnostics (Basel) 11(138):1391. 10.3390/diagnostics13081391 10.3390/diagnostics13081391 PMC10137254 37189492

[17] Ben Bouallègue F, Tabaa YA, Kafrouni M et al (2017) Association between textural and morphological tumor indices on baseline PET-CT and early metabolic response on interim PET-CT in bulky malignant lymphomas. Med Phys 44:4608–4619. 10.1002/mp.12349 28513853 10.1002/mp.12349

[18] Rodríguez Taroco MG, Cuña EG, Pages C et al (2021) Prognostic value of imaging markers from 18FDG-PET/CT in paediatric patients with Hodgkin lymphoma. Nucl Med Commun 42:306–314. 10.1097/MNM.0000000000001337 33306628 10.1097/MNM.0000000000001337

[19] Lue K-H, Wu Y-F, Liu S-H et al (2019) Prognostic value of pretreatment Radiomic features of 18F-FDG PET in patients with Hodgkin Lymphoma. Clin Nucl Med 44:e559–e565. 10.1097/RLU.0000000000002732 31306204 10.1097/RLU.0000000000002732

[20] Milgrom SA, Elhalawani H, Lee J, A PET Radiomics Model to Predict Refractory Mediastinal Hodgkin Lymphoma et al (2019) Sci Rep 9:1322. 10.1038/s41598-018-37197-z 10.1038/s41598-018-37197-z PMC6361903 30718585

[21] Sollini M, Kirienko M, Cavinato L et al (2020) Methodological framework for radiomics applications in Hodgkin’s lymphoma. Eur J Hybrid Imaging 4:9. 10.1186/s41824-020-00078-8 34191173 10.1186/s41824-020-00078-8 PMC8218114

[22] Zwanenburg A (2019) Radiomics in nuclear medicine: robustness, reproducibility, standardization, and how to avoid data analysis traps and replication crisis. Eur J Nucl Med Mol Imaging 46:2638–2655. 10.1007/s00259-019-04391-8 31240330 10.1007/s00259-019-04391-8

[23] Abenavoli EM, Linguanti F, Anichini M et al (2024) Texture analysis of 18F-FDG PET/CT and CECT: prediction of refractoriness of Hodgkin lymphoma with mediastinal bulk involvement. Hematol Oncol 42(2):e3261. 10.1002/hon.3261 38454623 10.1002/hon.3261

[24] Boellaard R, Delgado-Bolton R, Oyen WJG et al (2015) FDG PET/CT: EANM procedure guidelines for tumour imaging: version 2.0. Eur J Nucl Med Mol Imaging 42:328–354. 10.1007/s00259-014-2961-x 25452219 10.1007/s00259-014-2961-x PMC4315529

[25] Werner-Wasik M, Nelson AD, Choi W et al (2012) What is the Best Way to Contour Lung tumors on PET scans? Multiobserver Validation of a gradient-based Method using a NSCLC Digital PET Phantom. Int J Radiat Oncol 82:1164–1171. 10.1016/j.ijrobp.2010.12.055 10.1016/j.ijrobp.2010.12.055 PMC3877699 21531085

[26] Dibble EH, Lara Alvarez AC, Truong M-T et al (2012) ^18^ F-FDG Metabolic Tumor Volume and Total Glycolytic Activity of Oral Cavity and OropharysquamousucellscancerCaaddingAvalue Valclinicalistagingtaging. J Nucl Med 53:709–715. 10.2967/jnumed.111.099531 22492732 10.2967/jnumed.111.099531

[27] Dean EA, Mhaskar RS, Lu H et al (2020) High metabolic tumor volume is associated with decreased efficacy of axicabtagene ciloleucel in large B-cell lymphoma. Blood Adv 4:3268–3276. 10.1182/bloodadvances.2020001900 32702097 10.1182/bloodadvances.2020001900 PMC7391155

[28] Dinapoli N, Alitto AR, Vallati M et al (2015) Moddicom: a complete and easily accessible library for prognostic evaluations relying on image features. In: 2015 37th Annual International Conference of the IEEE Engineering in Medicine and Biology Society (EMBC).; IEEE, Milan, pp 771–774 10.1109/EMBC.2015.7318476 26736376

[29] Moddicom website https://github.com/kbolab/moddicom. Accessed 14 July 2022

[30] R Core Team. R: A language and environment for statistical computing. R Foundation for Statistical Computing, Vienna, Austria (2020) https://www.R-project.org/

[31] Zwanenburg A, Vallières M, Abdalah MA et al (2020) The image Biomarker Standardization Initiative: standardized quantitative Radiomics for High-Throughput Image-based phenotyping. Radiology 295:328–338. 10.1148/radiol.2020191145 32154773 10.1148/radiol.2020191145 PMC7193906

[32] Orlhac F, Eertink JJ, Cottereau A-S et al (2022) A guide to ComBat harmonization of imaging biomarkers in Multicenter studies. J Nucl Med 63:172–179. 10.2967/jnumed.121.262464 34531263 10.2967/jnumed.121.262464 PMC8805779

[33] Kallergi M, Georgakopoulos A, Lyra V, Chatziioannou S (2022) Tumor size measurements for Predicting Hodgkin’s and Non-hodgkin’s Lymphoma response to treatment. Metabolites 12:285. 10.3390/metabo12040285 35448472 10.3390/metabo12040285 PMC9024990

[34] Weber WA (2009) Assessing Tumor Response to Therapy. J Nucl Med 50. 10.2967/jnumed.108.057174.:1S-10S 10.2967/jnumed.108.057174 19380403

[35] Eertink JJ, van de Brug T, Wiegers SE et al (2022) 18F-FDG PET baseline radiomics features improve the prediction of treatment outcome in diffuse large B-cell lymphoma. Eur J Nucl Med Mol Imaging 49:932–942. 10.1007/s00259-021-05480-3 34405277 10.1007/s00259-021-05480-3 PMC8803694

[36] El-Galaly TC, Villa D, Gormsen LC et al (2018) FDG-PET/CT in the management of lymphomas: current status and future directions. J Intern Med 284:358–376. 10.1111/joim.12813 29989234 10.1111/joim.12813

[37] Carles M, Torres-Espallardo I, Alberich-Bayarri A et al (2017) Evaluation of PET texture features with heterogeneous phantoms: complementarity and effect of motion and segmentation method. Phys Med Biol 62:652–668. 10.1088/1361-6560/62/2/652 28033121 10.1088/1361-6560/62/2/652

[38] Pyradiomics Website https://pyradiomics.readthedocs.io/en/latest/features.html. Accessed 14 July 2022

[39] Meignan M, Cottereau A-S, Specht L, Mikhaeel NG (2021) Total tumor burden in lymphoma– an evolving strong prognostic parameter. Br J Radiol 94:20210448. 10.1259/bjr.20210448 34379496 10.1259/bjr.20210448 PMC8553180

[40] Jiang C, Li A, Teng Y et al (2022) Optimal PET-based radiomic signature construction based on the cross-combination method for predicting the survival of patients with diffuse large B-cell lymphoma. Eur J Nucl Med Mol Imaging 49(8):2902–2916. 10.1007/s00259-022-05717-9 35146578 10.1007/s00259-022-05717-9

[41] Ortega C, Eshet Y, Prica A et al (2023) Combination of FDG PET/CT Radiomics and Clinical Parameters for Outcome Prediction in Patients with Hodgkin’s Lymphoma. Cancers (Basel).; 30;15(7):2056. 10.3390/cancers15072056 10.3390/cancers15072056 PMC10093084 37046717

[42] Zhou Y, Zhu Y, Chen Z et al (2021) Radiomic features of 18F-FDG PET in Hodgkin Lymphoma are Predictive of outcomes. Contrast Media Mol Imaging 222021:6347404. 10.1155/2021/6347404 10.1155/2021/6347404 PMC8629643 34887712

[43] Driessen J, Zwezerijnen GJC, Schöder H et al (2023) Prognostic model using 18F-FDG PET radiomics predicts progression-free survival in relapsed/refractory Hodgkin lymphoma. Blood Adv 7(21):6732–6743. 10.1182/bloodadvances.2023010404 37722357 10.1182/bloodadvances.2023010404 PMC10651466

