# Supplementary material for: The predictive power of 18F-FDG PET/CT two-lesions radiomics and conventional models in classical Hodgkin’s Lymphoma: a comparative retrospectively-validated study
Source: Ann Hematol. 2025 Jan 14;104(1):641–51. doi: 10.1007/s00277-025-06190-8 (PMC11868178; doi:10.1007/s00277-025-06190-8)
Supplement: Supplementary file 1 — Supplementary Material 1 [file 277_2025_6190_MOESM1_ESM.docx]

**Supplemental TABLE 1.** Acquisition settings and reconstruction protocols of the two scanners employed

| Acquisition Protocol | | Scanner_1 | Scanner_2 |  |
| --- | --- | --- | --- | --- |
| CT | kV | 120 | |  |
|  | mAs | 40 - 50 | |  |
|  | Image matrix size | 512 x 512 | |  |
|  | Image pixel size (mm) | 3.5 x 3.5 0.97 x 0.97 | |  |
|  | Image slice thickness (mm) | 5 3 | |  |
|  | Range | from skull-base to mid-thighs | |  |
| PET | Reconstruction method | 3D-LOR-RAMLA  No PSF  No TOF | 3D-OSEM  PSF  TOF |  |
|  | Iterations | 3 | 2 |  |
|  | Subsets | 33 | 21 |  |
|  | Voxel size (mm^3^) | 4 x 4 x 4 | 3.2 x 3.2 x 5 |  |
|  | Filters applied | Gaussian filter 5 mm | Gaussian filter 2 mm |  |
|  | Image matrix size | 128 x 128 | 400 x 400 |  |
|  | Range | from skull-base to mid-thighs | |  |
|  | Minutes per bed position | 3 | 2.5 | |
| CT: Computed Tomography; LOR-RAMLA: Line of response-row-action maximum likelihood algorithm; OSEM: Ordered subset expectation maximization; PET: Positron Emission Tomography; PSF: Point-spread-function; Scanner_1: PHILIPS Gemini GXL; Scanner_2: SIEMENS Biograph mCT; TOF: Time-of-flight. | | | |  |

**Supplemental TABLE 2.** Radiomic features extracted from Lesion_A and Lesion_B through Moddicom software.

| IBSI* Class | Features | | | |
| --- | --- | --- | --- | --- |
| *Aggregation method* | *Averaged* | *2.5 D (direction merged)* | *Merged* | *2.5 D, Merged* |
| Morphological | F_morph.surface  F_morph.volume  F_morph.av  F_morph.comp.1  F_morph.comp.2  F_morph.sph.dispr  F_morph.sphericity  F_morph.asphericity  F_morph.com  F_morph.pca.major  F_morph.pca.minor  F_morph.pca.least  F_morph.pca.elongation  F_morph.pca.flatness |  |  |  |
| Intensity-based statistical | F_stat.mean  F_stat.var  F_stat.skew  F_stat.kurt  F_stat.median  F_stat.min  F_stat.10thpercentile  F_stat.90thpercentile  F_stat.max  F_stat.iqr  F_stat.range  F_stat.mad  F_stat.rmad  F_stat.energy  F_stat.rms |  |  |  |
| Intensity-histogram | F_stat.entropy  F_stat.uniformity |  |  |  |
| GLCM^†^  Presents the  number of times that two intensity levels have occurred in two pixels with specific distance | F_cm.joint.max  F_cm.joint.avg  F_cm.joint.var  F_cm.joint.entr  F_cm.diff.avg  F_cm.diff.var  F_cm.diff.entr  F_cm.sum.avg  F_cm.sum.var  F_cm.sum.entr  F_cm.energy  F_cm.contrast  F_cm.dissimilarity  F_cm.inv.diff  F_cm.inv.diff.norm  F_cm.inv.diff.mom  F_cm.inv.diff.mom.norm  F_cm.inv.var  F_cm.corr  F_cm.auto.corr  F_cm.clust.tend  F_cm.clust.shade  F_cm.clust.prom  F_cm.info.corr.1  F_cm.info.corr.2 | F_cm_2.5D.joint.max  F_cm_2.5D.joint.avg  F_cm_2.5D.joint.var  F_cm_2.5D.joint.entr  F_cm_2.5D.diff.avg  F_cm_2.5D.diff.var  F_cm_2.5D.diff.entr  F_cm_2.5D.sum.avg  F_cm_2.5D.sum.var  F_cm_2.5D.sum.entr  F_cm_2.5D.energy  F_cm_2.5D.contrast  F_cm_2.5D.dissimilarity  F_cm_2.5D.inv.diff  F_cm_2.5D.inv.diff.norm  F_cm_2.5D.inv.diff.mom  F_cm_2.5D.inv.diff.mom.norm  F_cm_2.5D.inv.var  F_cm_2.5D.corr  F_cm_2.5D.auto.corr  F_cm_2.5D.clust.tend  F_cm_2.5D.clust.shade  F_cm_2.5D.clust.prom  F_cm_2.5D.info.corr.1  F_cm_2.5D.info.corr.2 | F_cm_merged.joint.max  F_cm_merged.joint.avg  F_cm_merged.joint.var  F_cm_merged.joint.entr  F_cm_merged.diff.avg  F_cm_merged.diff.var  F_cm_merged.diff.entr  F_cm_merged.sum.avg  F_cm_merged.sum.var  F_cm_merged.sum.entr  F_cm_merged.energy  F_cm_merged.contrast F_cm_merged.dissimilarity  F_cm_merged.inv.diff  F_cm_merged.inv.diff.norm  F_cm_merged.inv.diff.mom  F_cm_merged.inv.diff.mom.norm  F_cm_merged.inv.var  F_cm_merged.corr  F_cm_merged.auto.corr  F_cm_merged.clust.tend  F_cm_merged.clust.shade  F_cm_merged.clust.prom  F_cm_merged.info.corr.1  F_cm_merged.info.corr.2 | F_cm.2.5Dmerged.joint.max  F_cm.2.5Dmerged.joint.avg  F_cm.2.5Dmerged.joint.var  F_cm.2.5Dmerged.joint.entr  F_cm.2.5Dmerged.diff.avg  F_cm.2.5Dmerged.diff.var  F_cm.2.5Dmerged.diff.entr  F_cm.2.5Dmerged.sum.avg  F_cm.2.5Dmerged.sum.var  F_cm.2.5Dmerged.sum.entr  F_cm.2.5Dmerged.energy  F_cm.2.5Dmerged.contrast  F_cm.2.5Dmerged.dissimilarity  F_cm.2.5Dmerged.inv.diff  F_cm.2.5Dmerged.inv.diff.norm F_cm.2.5Dmerged.inv.diff.mom  F_cm.2.5Dmerged.inv.diff.mom.norm  F_cm.2.5Dmerged.inv.var  F_cm.2.5Dmerged.corr  F_cm.2.5Dmerged.auto.corr  F_cm.2.5Dmerged.clust.tend  F_cm.2.5Dmerged.clust.shade  F_cm.2.5Dmerged.clust.prom  F_cm.2.5Dmerged.info.corr.1  F_cm.2.5Dmerged.info.corr.2 |
| GLRLM^‡^  Presents the  length of consecutive pixels having the same intensity | F_rlm.sre  F_rlm.lre  F_rlm.lgre  F_rlm.hgre  F_rlm.srlge  F_rlm.srhge  F_rlm.lrlge  F_rlm.lrhge  F_rlm.glnu  F_rlm.glnu.norm  F_rlm.rlnu  F_rlm.rlnu.norm  F_rlm.r.perc  F_rlm.gl.var  F_rlm.rl.var  F_rlm.rl.entr | F_rlm_2.5D.sre  F_rlm_2.5D.lre  F_rlm_2.5D.lgre  F_rlm_2.5D.hgre  F_rlm_2.5D.srlge  F_rlm_2.5D.srhge  F_rlm_2.5D.lrlge  F_rlm_2.5D.lrhge  F_rlm_2.5D.glnu  F_rlm_2.5D.glnu.norm  F_rlm_2.5D.rlnu  F_rlm_2.5D.rlnu.norm  F_rlm_2.5D.r.perc  F_rlm_2.5D.gl.var  F_rlm_2.5D.rl.var  F_rlm_2.5D.rl.entr  F_rlm_25D_merged.dfge |  | F_rlm.2.5Dmerged.sre  F_rlm.2.5Dmerged.lre  F_rlm.2.5Dmerged.lgre  F_rlm.2.5Dmerged.hgre  F_rlm.2.5Dmerged.srlge  F_rlm.2.5Dmerged.srhge F_rlm.2.5Dmerged.lrlge  F_rlm.2.5Dmerged.lrhge  F_rlm.2.5Dmerged.glnu  F_rlm.2.5Dmerged.glnu.norm  F_rlm.2.5Dmerged.rlnu  F_rlm.2.5Dmerged.rlnu.norm  F_rlm.2.5Dmerged.r.perc  F_rlm.2.5Dmerged.gl.var  F_rlm.2.5Dmerged.rl.var  F_rlm.2.5Dmerged.rl.entr |
| GLSZM^§^  Considers the size of homogeneous  zones in every dimension | F_szm.sze  F_szm.lze  F_szm.lgze  F_szm.hgze  F_szm.szlge  F_szm.szhge  F_szm.lzlge  F_szm.lzhge  F_szm.glnu  F_szm.glnu.norm  F_szm.zsnu,  F_szm.zsnu.norm  F_zsm.z.perc  F_szm.gl.var  F_szm.zs.var  F_szm.z.entr | F_szm_2.5D.sze  F_szm_2.5D.lze  F_szm_2.5D.lgze  F_szm_2.5D.hgze  F_szm_2.5D.szlge  F_szm_2.5D.szhge  F_szm_2.5D.lzlge  F_szm_2.5D.lzhge  F_szm_2.5D.glnu  F_szm_2.5D.glnu.norm  F_szm_2.5D.zsnu  F_szm_2.5D.zsnu.norm  F_zsm_2.5D.z.perc  F_szm_2.5D.gl.var  F_szm_2.5D.zs.var  F_szm_2.5D.z.entr |  |  |
| *: Image biomarker standardization initiative  ^†^: Grey Level Co-occurrence Matrix-based features.  ^‡^: Grey Level Run Length Matrix-based features.  ^§^: Grey Level Size Zone Matrix-based features. | | | | |

**Supplemental TABLE 3.** Clinical, radiomic, conventional PET/CT and combined models’ significance for PFS prediction in patients with overlaping Lesion_A and Lesion_B (n. 41)

| Models | AUC [95% CI] | | |
| --- | --- | --- | --- |
|  | **C-index** | **95%CI** |  |
| C | **75,2%** | **[56,1;94,3]*** |  |
| R | **51,4%** | **[24,5;78,3]** |  |
| P | **46,7%** | **[23,1;70,2]** |  |
| C+R | **73,3%** | **[56,5;90,1]*** |  |
| C+P | **78,1%** | **[58,9;97,2]*** |  |
| R+P | **52,4%** | **[25,6;79,1]** |  |
| C+R+P | **72,4%** | **[55,5;89,2]*** |  |
| C: clinical model (stage and IPS); P: conventional PET/CT model (D_max_ of Lesion_A and TMTV); R: radiomic model (*F_cm.corr* from Lesion_B); *: significant models. | | |  |

**Supplemental TABLE 4.** Clinical, radiomic, conventional PET/CT and combined models’ significance for DS prediction in patients with overlapping Lesion_A and Lesion_B (n. 41)

| Models | AUC [95% CI] | |  |
| --- | --- | --- | --- |
|  | **AUC** | **95%CI** | |
| C | **68,5%** | **[51,6;85,4]*** | |
| R | **64,5%** | **[43,2;85,7]** | |
| P | **71,4%** | **[49,7;93]** | |
| C+R | **72,2%** | **[54,5;89,8]*** | |
| C+P | **73,0%** | **[54,5;91,3]*** | |
| R+P | **81,5%** | **[66,7;96,1]*** | |
| C+R+P | **81,5%** | **[66,6;96,2]*** | |
| C: clinical model (stage and IPS); P: conventional PET/CT model (D_max_ of Lesion_A, SUV_max_ of Lesion_B and TMTV); R: radiomic model (*F_szm.glnu* and *F_rlm.2.5D.rlnu* from Lesion_B and *F_szm.glnu* from Lesion_A); *: significant models. | | |  |
